# A Systematic Review of Online Speech Therapy Systems for Intervention in Childhood Speech Communication Disorders

**DOI:** 10.3390/s22249713

**Published:** 2022-12-11

**Authors:** Geertruida Aline Attwell, Kwabena Ebo Bennin, Bedir Tekinerdogan

**Affiliations:** Wageningen University and Research, 6706 KN Wageningen, The Netherlands

**Keywords:** automated speech therapy, online speech therapy, telepractice software, communication disorder

## Abstract

Currently, not all children that need speech therapy have access to a therapist. With the current international shortage of speech–language pathologists (SLPs), there is a demand for online tools to support SLPs with their daily tasks. Several online speech therapy (OST) systems have been designed and proposed in the literature; however, the implementation of these systems is lacking. The technical knowledge that is needed to use these programs is a challenge for SLPs. There has been limited effort to systematically identify, analyze and report the findings of prior studies. We provide the results of an extensive literature review of OST systems for childhood speech communication disorders. We systematically review OST systems that can be used in clinical settings or from home as part of a treatment program for children with speech communication disorders. Our search strategy found 4481 papers, of which 35 were identified as focusing on speech therapy programs for speech communication disorders. The features of these programs were examined, and the main findings are extracted and presented. Our analysis indicates that most systems which are designed mainly to support the SLPs adopt and use supervised machine learning approaches that are either desktop-based or mobile-phone-based applications. Our findings reveal that speech therapy systems can provide important benefits for childhood speech. A collaboration between computer programmers and SLPs can contribute to implementing useful automated programs, leading to more children having access to good speech therapy.

## 1. Introduction

Young children judge each other based on their communication skills, and therefore, a communication disorder can harm someone’s social status at a young age [1]. Children enrolled in therapy before the age of five experience more positive outcomes than children that enroll after this age [2]. Even when access to a speech–language pathologist (SLP) is possible, SLPs often struggle to manage their therapy plans for each of their clients. In general, an SLP works with a client weekly and has a session with them for one hour [3]. During the session, the SLP gives the client several exercises and registers the client’s performance and progress. At the end of such a session, the therapist is supposed to generate a personalized therapy plan. Going through a routine of working with several clients a day makes it hard for the SLP to provide a personal therapy plan at the end of the session [3]. The task of an SLP is broad and does not only entail doing exercises with the patient, as is thought a lot. As the American Speech–Language-Hearing Association (ASHA) states: “Speech–language pathologists (SLPs) work to prevent, assess, diagnose, and treat speech, language, social communication, cognitive-communication, and swallowing disorders in children and adults” [4].

Previous research mentions the positive impact that the usage of Information and Communication Technologies (ICT) can offer during therapy [5]. Children in developed countries are coming in contact with digitalization more frequently and quickly embrace the online process [6]. Additionally, in interventions for children with communication impairments, the use of ICT is increasing, and telepractice is a rapidly evolving field, which is resulting in the development of several online speech therapy (OST) systems [6]. The appeal of digital technology to children is seen as one of its benefits, together with the accessibility it provides in simplifying the process of matching clients with clinical expertise [6].

Currently, there is a worldwide shortage of SLPs [7]. This results in approximately 70% of the SLPs having waiting lists, including in developed countries, such as The Netherlands [7,8]. Currently, not all children that need intervention for their communication impairment have access to a therapist. However, with software systems’ availability in developed countries, speech therapy can be accessible to all without physical contact. This paper aims to provide a systematic literature review (SLR) to systematically identify, analyze and describe the state-of-the-art advances in OST systems for communication disorders. This study evaluates the current state of the art regarding OST systems for children with communication disorders which have assessment and/or an intervention functions or assist the SLP in another tailored way.

The rest of this article is organized as follows: Section 2 provides some brief background information on communication disorders, OST, speech recognition, machine learning (ML), and related works. Section 3 explains the SLR methodology. Section 4 presents the results of the SLR on OST Systems. Section 5 covers the discussion. Finally, Section 6 presents the conclusions.

## 2. Background and Related Works

### 2.1. Communication Disorders

According to the American Speech–Language-Hearing Association (ASHA), a communication disorder is “an impairment in the ability to receive, send, process, and comprehend concepts or verbal, nonverbal and graphic symbol systems. A communication disorder may be evident in the processes of hearing, language, and/or speech” [4]. Among communication disorders, the ASHA distinguishes between four variations, namely, (a) speech disorders affecting processing or production of the sounds of a language (phonology and motor planning and executive); (b) language disorders affecting the comprehension or production of semantics, morpho-syntax or discourse; (c) hearing disorders; and (d) central auditory processing disorders. A speech disorder is a problem with fluency, voice and how a person says speech/sounds [2]. A speech disorder is also referred to as a phonological disorder or a speech sound disorder (SSD). A SSD should not be confused with a communication disorder. Speech disorders are communication disorders that disrupt everyday speech [9]. The ASHA states that this can be due to an impairment in the articulation of speech sounds, fluency and/or voice [4].

### 2.2. Telepractice and OST

The ASHA defines telepractice as the application of telecommunications technology to the delivery of Speech–Language pathology and professional audiology services at a distance by linking clinician to client or clinician for assessment, intervention and consultation [4]. Throughout the years, several interactive tools have been set up to reduce the time required from the SLP [10]. However, the term telecommunications technology is rather broad, and the boundaries are not always clear. Computers in speech therapy are also referred to as computer-based speech therapy (CBST) [7]. The following definition of computer-based speech therapy is given by Furlong et al. [7] (p. 51): “A CBST program is a software offering predefined therapy tasks inclusive of instructional features (e.g., an animated talking tutor, the use of synthesized speech to provide models or instructions), motivational features (e.g., the use of animations, game-based activities) and quantitative features (e.g., the tracking of performance within and across therapy sessions), operating from a personal computer”. According to Furlong and colleagues [7], this does not entail mobile applications and wireless devices, such as tablets. However, as mentioned in Section 1, one of the advantages of telepratice is accessibility [6]; as a result, some of the developed OST systems are focused on mobile phones and tablets, as they can quickly provide access through mobile connectivity; therefore, this review also considers these systems. Chen et al. [11] referred to an interactive speech therapy computer program with the term virtual speech therapist (VST). Programs are designed to help learn a non-native language or developed to help people whose speech abilities are already in the normal range, and thus, they were not considered to be VST programs by Chen et al. [11].

### 2.3. Speech Recognition

Automatic speech recognition (ASR) has been a hot topic in research for more than half a century [12]. ASR can be described as the transformation of the acoustic microstructure of a speech signal into its implicit phonetic macro-structure. In other words, it is speech-to-text conversion where the output is digital text corresponding with the recognized speech [13]. The general architecture of a speech recognition system was provided by Padmanabhan and Premkumar [14]. When the speech recognition system receives the input signal, it gets pre-processed by the front end, sending spectral-like features to the decoder. The decoder contains a phone likelihood estimator, which scores each phone’s likelihood [14]. Ghai and Singh [13] distinguished four different ASR approaches, of which the first one is the acoustic-phonetic approach. According to this approach, discovered by Hemdal and Hughes in 1967, there is a fixed number of phonetic units that can be distinguished in each language. Each unit has its own components, which can be recognized by its properties, such as nasality. A second approach, the connectionist approach, looks at patterns of units instead of individual units. The dominant approach in ASR is the so-called pattern recognition approach [13], which is executed with a mathematical framework. The final approach, the knowledge-based approach, can be seen as a mix of acoustic–phonetic and pattern recognition approaches. In general, an ASR system has two phases: the training phase and the recognition phase [13].

### 2.4. Machine Learning

Machine learning (ML) is defined as the study of computer algorithms that improve automatically through experience and by the use of data. Three types of machine learning can be distinguished: supervised learning, unsupervised learning and reinforcement learning. In supervised ML, the ML algorithm is trained with a labeled dataset, and an output response or class for each input data vector is given. With unsupervised ML, the ML algorithm is expected to learn the patterns in the unlabeled input dataset by itself without any feedback, for example, from an speech language pathologist (SLP) [14]. Finally, in reinforcement learning, an agent learns the best actions possible in an interactive environment in order to attain the defined goals.

Back in 1970, a wide variety of different architectures and approaches were tried for speech recognition. Many of these were relatively informal and weak and were feasible for only a few specially selected cases. In recent years, approaches based on hidden Markov models (HMMs) have become more dominant [15]. Hidden Markov models are based on a rather complex mathematical theory and are commonly generated by training a large corpus of actual speech data. Another popular method is neural networks. Neural networks consist of units connected by links [15]. In artificial neural networks (ANN), units are trained using input–output datasets presented to the network. After the training procedure, the network generates proper outcomes when tested with parallel datasets, which means that the network recognizes the patterns it was trained with during the training process [16].

### 2.5. Related Work

Chen et al. [11] published a systematic review on computer-based speech therapy systems focused on speech disorders, including hearing impairments and aphasia. The review mainly focused on the technological details of CBSTs. The results suggest that such systems can result in a higher engagement from the clients throughout the therapy. A challenge of this study is the heterogeneity of several critical aspects addressed in the review, such as types of disorders, outcome measures, and study design types [17]. Furthermore, Furlong et al. [7] conducted a systematic literature review to evaluate the efficacy of CBST for children with an SSD. The preliminary conclusion from the study is that CBST can benefit children with dysarthria, articulation impairment, phonological impairment, and SSDs associated with hearing impairment [7]. For their study, they only included 14 studies, and all the included studies were from 2008 or before, which led to the exclusion of any relevant studies after 2008. Additionally, speech recognition technologies such as ML used in some of these systems have not yet been discussed [7]. We provide a more up-to-date and extensive literature review that also takes conference papers into consideration, thereby providing us with many OST systems that otherwise would be overlooked. Seven out of the 20 studies reviewed in Chen et al. [11] and six out of the 14 studies reviewed by Furlong et al. [7] reported on OST systems designed to provide speech production feedback to the user. Such feedback was provided mainly in the form of visual feedback. Noteworthy is that neither one of the reviews included mobile technology [18].

## 3. Research Methodology

We conducted a systematic literature review (SLR), a well-defined method to identify, evaluate and interpret all relevant studies regarding a particular topic [19]. This method helps summarize existing evidence concerning a technology, in our case, already existing or proposed OST systems. The review protocol defines the research questions, search strategy to identify relevant literature, study selection criteria, quality assessment and methodology for extracting and synthesizing information from relevant papers [19]. We provide more details on the review protocol in the remainder of this section.

### 3.1. Research Questions

As stated before, no systematic review is provided in the literature on how automated speech therapy systems support SLPs and the challenges and obstacles of designing and adopting OST systems. Thus, our main goal was to summarize the evidence related to how OST systems are designed and evaluated. Hence, we answer the following two main questions with their respective sub-questions:

**RQ1**: What are the existing OST systems?

- RQ1.1: For which goals/context have OST Systems been developed?
- RQ1.2: What are the features of the OST systems?
- RQ1.3: For which target groups have OST systems been used?
- RQ1.4: What are the adopted architecture designs of the OST systems?
- RQ1.5: What are the adopted ML approaches in these OST systems?
- RQ1.6: What are the properties of the software used for these systems?

**RQ2**: How efficacious are OST systems?

- RQ2.1: Which evaluation approaches have been used to assess the efficacy of the OST systems?
- RQ2.2: Which performance metrics have been used to gauge the efficacy of the OST systems?

### 3.2. Search Strategy

A systematic selection process involving the scanning of titles, abstracts and full texts was completed. Articles and conference papers written in English reporting a trial of an OST program for SSD treatment in children were included.

**Figure 1:**
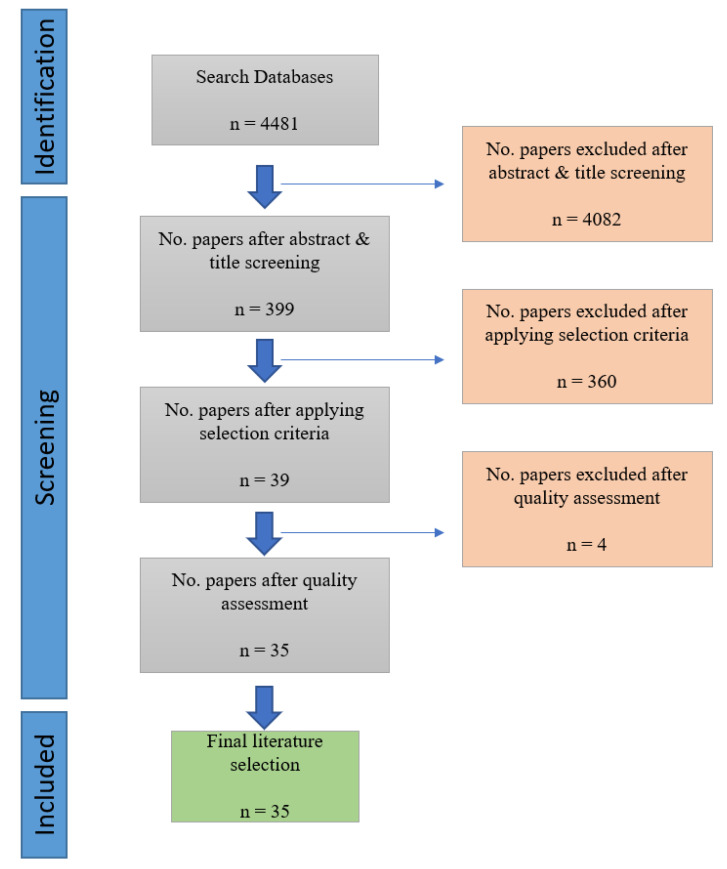
Flowchart search strategy.

A flowchart of the search strategy can be seen in Figure 1.

#### 3.2.1. Search Scope

We limited our search to two dimensions: the publication period and electronic data sources. We limited our search to papers published from January 2005 to February 2021. The start date of January 2005 was due to a rapid change in technology, as there was a risk that articles published before this date would no longer be relevant. The databases used for the search were Scopus; Web of Science; IEEE; and ACM. Papers were selected based on their titles and abstracts. Papers considered during this search process were journal papers and conference papers. Table 1 shows the number of papers obtained from each electronic data source.

#### 3.2.2. Search Method

We used an automatic search as our search method and used the following search string:

("speech and language assessment" OR "speech therapy" OR "speech sound disorder*" OR "voice disorder*" OR "speech and language disorder*" OR "speech disorder" OR "speech delay" AND "tool*" OR "information system*" OR "software" OR "expert system*" OR "artificial intelligence" OR "online system" OR "computer-based")

The query was tested first with an extra compartment, namely: AND "child*". However, it was noticed that some critical papers would not be selected when this was added to the query.

### 3.3. Study Selection Criteria

Study selection criteria were set up to determine which studies should be excluded from the review. The overall study selection criteria that we used are displayed in (Table 2):

Following the application of the selection criteria, 39 articles were kept and considered for quality assessment.

### 3.4. Study Quality Assessment

In addition to the study selection criteria, the primary studies’ quality was assessed with the criteria set up by Kitchenham and Charters [19]. Table 3 presents the quality checklist. We used a three-point scale to assign scores (yes = 2, somewhat = 1, no = 0) to each criterion. An overview of the score of each paper can be found in Appendix A. The maximum score a paper could obtain was 14. Papers with a score of 7 or lower were left out.

### 3.5. Data Extraction and Monitoring

To extract data, the full texts of the 35 selected studies were read. A data extraction form was created to collect all the information needed to address the research questions and the study quality criteria. This form can be found in Appendix B. The data extraction was done with the help of a Microsoft Excel spreadsheet which is freely available online: https://bit.ly/3Fv7qDp (accessed on 1 November 2022). Data from the included studies were extracted by two authors and verified by the third author.

### 3.6. Data Synthesis and Reporting

When performing an SLR, the extracted data should be synthesized in a manner suitable for answering the questions that an SLR seeks to answer [19]. After the quality assessment, 35 of the 39 articles made it to the final literature selection; these articles are shown in Table 4 below.

## 4. Results

In this section, we synthesize the SLR results and provide the answers to the identified research questions. In total, the selected papers (Table 4) discussed 35 systems. Study AA was a further improvement of the system set up in study J. Study DD was a further improvement of the system set up in study W. Study AA and study DD provided further experimental validation of their previous studies. Given that both studies AA and DD also introduced new elements, we considered them in the primary studies.

### 4.1. For Which Goals/Context Have OST Systems Been Developed?

**Figure 2:**
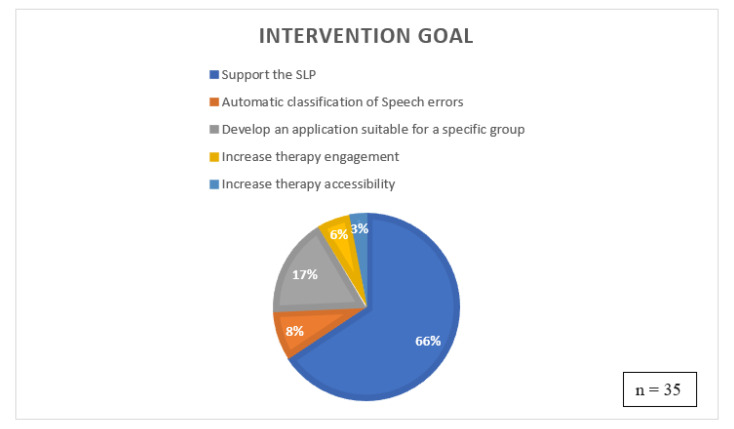
Intervention goal OST systems.

Figure 2 shows the distribution of the intervention goals of each study. The categorization of intervention goal for each study can be tricky, as some of the indicated intervention goals can overlap. For example, a study that uses automatic classification of speech errors can also be developed to support the SLP. The following classification is based on what each paper mentions as main intervention goal for the development of the system. The majority of the OST systems’ main goal was to support the SLP in his/her current activities (66%). The majority of the reviewed studies discussed developing a system intending to assist the SLP with their work and at the same time provide clients with the possibility to continue their practice without the constant need for an SLP present. Studies G, I, L, N, GG and II (17%) focused on building an application suitable for a specific target group, such as children with cerebral paresis. Studies C, EE and FF (8%) developed a system to automatically classify speech errors in clients’ spoken text. One could argue that this falls under the category of "Support the SLP", but in reality, diagnosing a client often happens in the first session and support is mainly needed during the follow-up sessions, which is why these three papers got their categorization "automatic classification of speech errors". Studies Q and T (6%) tried to set up a system specifically focused on maintaining the child’s attention during the therapy session. Finally, study B (3%) aimed to increase accessibility by setting up a game environment. Increasing accessibility was also mentioned in other studies, but rather as a consequence rather than as a study’s primary goal.

**Figure 3:**
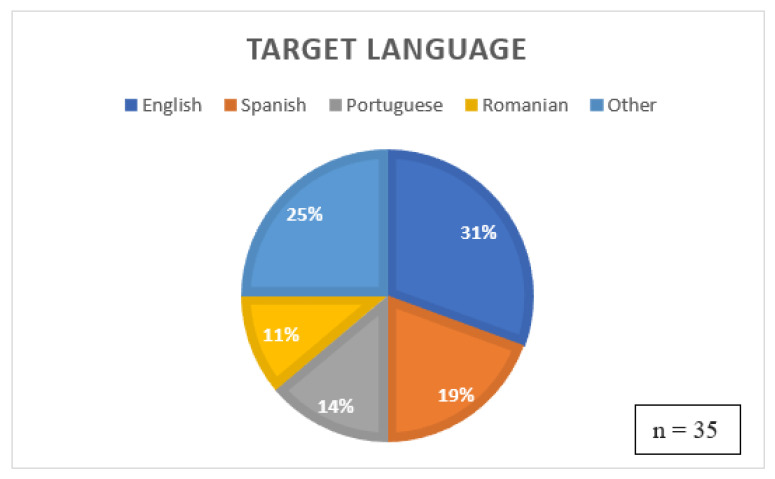
Overview of target languages of OST Systems.

Four of the thirty-five studies described “people” as their target group. However, these studies (i.e., F, J, P and II) included both adults and children. Thus, these studies were also considered and not removed from the list, as the systems discussed might also be beneficial for children. The distribution of the target language of the reviewed systems is portrayed in Figure 3. It is evident from the figure that most systems were designed with a focus on the English language. Spanish, Portuguese and Romanian were the next most common languages apart from English.

### 4.2. What Are the Features of the OST Systems?

To fully understand the functionality of OST systems, it is essential to know which features they have. Features can be defined as user-visible characteristics. In total, 14 common features among the 35 studies were identified. The explanations of the features are described below based on the explanations given in the primary studies.

**Figure 4:**
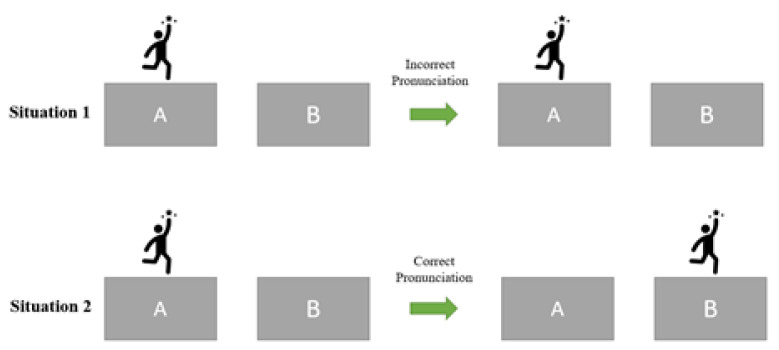
Representation of visual feedback.

- Audio feedback is audio output from a system that informs the user whether he or she is performing well.
- Emotion screening: The system considers the client’s emotions throughout the session, for example, by measuring the facial expressions with a face tracker or the system asking the child how he or she feels or to rate the child’s feelings.
- Error detection: Identification of errors made through speech analysis algorithms by analyzing produced vowels and consonants individually (Parnandi et al., 2013 [39]).
- Peer-to-peer feedback is a feature that enables multiple clients to participate with each other in an exercise. Peers can provide feedback to each other’s performance in terms of understandability, the volume of sound and so on, depending on the exercise’s context and scope.
- Speech recognition, also known as speech-to-text or automatic speech recognition, is a feature that enables a program to convert human speech into a written format.
- Recommendation strategy: A feature that provides suggestions for helpful follow-up exercises and activities that can be undertaken by the SLP based on the correctness of pronunciation (Franciscatto et al., 2021 [1]).
- Reporting: Providing statistical reports about the child’s progress and the level of performance during the session.
- Text-to-speech: A feature that can read digital text on a digital device aloud.
- Textual feedback is textual output from a system that shows the user whether he performs well. For example, when the word is pronounced correctly, the text “Correct Answer” appears, whereas if the word is mispronounced, the text “Incorrect Answer” appears, possibly with an explanation of why it is incorrect.
- User data management: Everything that has to do with keeping track of the personal data of clients, such as account names and age.
- User voice recorder: A feature that provides the option to record the spoken text by the clients. The recorded voice can be played back by, for example, the client, the SLP or other actors such as a teacher or parent.
- A virtual 3D model aids in viewing the correct positioning of the lips, language and teeth for each sound (Danubianu, 2016).
- Visual feedback is visual output from a system, such as a video game, that shows the user if he or she is performing well or not. For example, a character only proceeds to the next level when the client has pronounced the word correctly. Visual feedback is the character’s movement from level A to level B, as illustrated in Figure 4.
- Voice commands are spoken words by the child that let the system act. For example, when a child says jump, the character in a game jumps.

The features and their corresponding studies are presented in Table 5. By examining the features in the table, we can observe that reporting is the most dominant feature. This was not unexpected, since two-third of the systems were made to support the SLP. A reporting feature provides the SLP a quick overview of the child’s progress and the mistakes made, saving much time for one-on-one interaction. The most repeatedly used report types are statistical data of properly practiced words and statistical data of overall activities during the therapy session. Most of the papers (25) do not discuss any reporting feature for their systems.

### 4.3. For Which Target Groups Have OST Systems Been Used?

An overview of the target disorders can be found in Table 6.

Table 6 presents an overview of the target communication disorders of the papers. During the literature analysis, we came across many different terms. For example, study H mentioned “speech disorder” as its target disorder, study J mentioned “speech impairments” and study P mentioned “speech impediments”. As shown in Table 6, 19 studies mentioned “speech disorder” as their target disorder, which is the majority. However, this term is rather broad. In study B, they mention that the 3D game environment was developed to meet the specific requirements of “language disordered” children, but did not clarify the type of disorder. They tested the system on a child who had a language disorder because of hearing impairment, but they do not mention if the final system was developed for this focus group. The system of study F provides tasks for both aphasia and speech disorders; however, aphasia is a non-speech disorder. Studies in the communication disorder groups did not further define the participants’ disorders (or the target disorder) regarding speech, language and hearing. This may have been due in part to the developed systems being generic SLP-assistance OSTs designed to simply support the SLP in client management.

### 4.4. What Are the Adopted Architecture Designs of the OST Systems?

In this analysis, we aimed to examine the studies that presented and discussed the adopted architectural patterns used in developing the OST systems. Fifteen of the thirty-five studies did not mention or discuss any architecture approach at all. The rest of the studies did, but the extent of details discussed differed a lot per study. Table 7 provides an overview of the adopted architectural patterns of those 20 studies that did discuss them. In the subsections below, we discuss each architecture found in the studies.

#### 4.4.1. Client–Server System

From Table 7, the most commonly adopted architecture approach was a client–server approach. Study D used a two-tier client–server architecture in which data mining was used to derive knowledge from the data. Study H described a software architecture containing a capture module and a service module. The first collects the client’s audio data and then revises the data, which the SLP eventually double-checks after it is checked by the service module. In study F, they created a virtual therapist in the application that provides the sound/word task with audiovisual cues and articulation. The client pronounces the sound/word, and the virtual therapist then sends the client’s audio response to the system server. The system server analyses the speech and detects mispronunciation. The detected errors are sent back to the application, which sends feedback to the client. The system in study L also has one module for the therapist, namely, the assessment module. In the assessment module, the SLP can view the clients and their scores, and the practice module is used by the client to do exercises. The system described in study P has a mobile application used by the client and a server that manages all the applications’ requests. All the data are stored in a central database. An Internet connection is needed from time to time to synchronize the client’s progress in the application with the central database. The server module of study U was threefold, as it comprises a management application program, a database server and a data transfer application. When the child is playing the game, its interactions are captured by the desktop client software and stored on a local open-source database. The therapist can review these interactions and send the data to the database server with the data transfer application’s help. Through an Internet connection, the therapist then has remote access to the data of each child. The application of study V has a multi-tier client–server architecture and provides remote administration of speech therapy. Through the user interface, the SLP can remotely manage therapy for his or her clients, create exercises and organize speech recordings, as this application is also where the speech analysis can be done. The client has access to the therapy session through a mobile application. Study DD showed a detailed architecture of the speech therapy system, but no additional explanation was provided in the other studies that presented a picture of the RA. The provided pictures showed the interaction between the SLP’s computer and the client’s mobile device. The expert system on the computer of the therapist selects exercises, and if the SLP agrees to them, they are sent to the child. The mobile device collects the child’s vocal production, and the results are sent to the monitor program installed on the SLP’s computer. The design of the online expert system in study HH is based on a system that is built from smaller subsystems, including the conditions for articulation disorders, phonological disorders, fluency disorders and language disorders. Study II discussed their architecture in the style of a block diagram. The application works with only audio input and lets the user know whether the word/sentence was pronounced correctly or not.

#### 4.4.2. Repository Pattern

The system’s interface in study T tests a client’s performance with the help of a database with correctly pronounced sounds. The game developed in study CC stores the speech input in an SQL database; the study mainly discusses the pre-processing, feature extraction and automatic speech recognition, but does not go into deep detail regarding the RA.

#### 4.4.3. Layered Approach

The system proposed in study S relies on a user interface, an expert system and a domain knowledge layer. Each layer interacts with the other and provides different services. The UI and services layer provide several functionalities for helping SLPs. The expert-system layer relies on two modules for performing the processes to interact with users and support the decision making of SLPs. The domain knowledge is managed in the last layer through ontologies, databases for monitoring and activities, standardized vocabularies and a clinical data repository. The interaction layer of the system in study X has two applications, one that the therapist can use to manage the exercises of the client and one to perform administrative tasks. The information captured in the interaction layer is sent to the expert system in the service layer, which contains the following modules: a web services module, a user management module, a report generation module and a module for speech recognition. The SPELTA system from study Y consists of different systems that all work with a knowledge base. The SPELTA-miner system in study Z is an expert system responsible for conducting machine learning, analyzing and generating therapy plans and educational content.

#### 4.4.4. Standalone System

The architecture design in study A was only textually described, and no figure/image was provided. The client practices pronunciation with the help of the training module.

#### 4.4.5. Pipe-and-Filter Architecture

In the systems from studies E and W, the modules are mainly designed for assisting the SLP. The two main components of the system are an intelligent system that is installed on the computer of the SLP and a mobile system used by the client. Between the SLP and the client itself, there is a personal relationship, and they can see each other through the home monitor program. The intelligent system contains a 3D model that analyses the client’s words, and these can be reviewed by the SLP. The data are transferred to the expert system, and it can offer suggestions regarding which exercises are most suitable for the child. It is a fuzzy expert system that is rule-based, which makes it easier for the SLP to understand. The proposed system in study FF consists of a tablet-based mobile application that records the child’s speech when he is talking during the exercises. The spoken words are assessed by the voice activity detector (VAD) in the speech recognition engine, which provides the assessment results to the SLP at the interface. At this interface, the SLP can also create and assign new exercises to clients and obtain an overview of each child’s progress.

### 4.5. What Are the Adopted Machine Learning Approaches in These OST Systems

**Figure 5:**
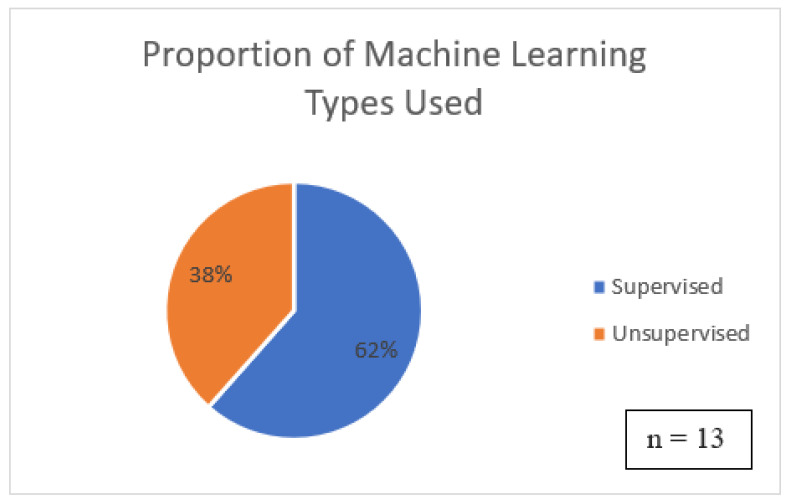
The proportion of ML types in the literature.

The majority of the papers did not discuss the usage of machine learning (ML). Only 13 of the 35 primary studies discussed which ML approach they used to develop the system. In all cases, the ML types could be broken down into either supervised or unsupervised. Semi-supervised and reinforcement learning were not mentioned in the papers. Figure 5 shows the distribution of the ML types.

Supervised learning is more commonly used than unsupervised learning because more clustering tasks are applied in OST Systems. The proportions of ML types are shown in Figure 5.

**Figure 6:**
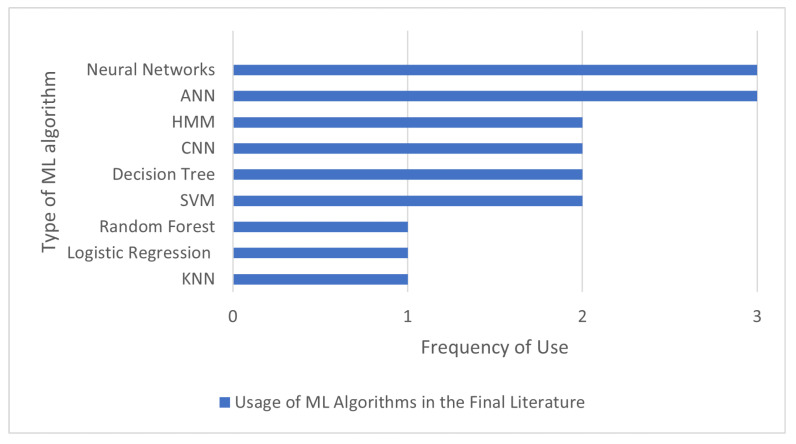
The number of times each algorithm appeared in the final literature.

Figure 6 shows the number of times each algorithm appeared in the final literature. The algorithms that each paper used are presented in Table 8. Study A used automatic speech recognition (ASR) as a tool to transfer the speech signal into a string of words. The words spoken by the client in the microphone are processed by a computer program that first extracts the spoken text features. These extracted features are then compared with the trained patterns. Natural language processing was used for the text-to-speech system. The text that is given as input comes out as synthetic voice output.

In study C, a convolutional neural network (CNN) was used to recognize the child’s words during the gameplay. The authors also tried other classification models for speech recognition, such as support vector machines and artificial neural networks. However, using the CNN model gave the lowest number of false negatives compared to the other models. In comparison with the other studies, the explanation was very extensive. The study also showed a representation of the 1D CNN architecture. To evaluate the children’s pronunciations, study E discussed the use of a fuzzy logic algorithm that assigns weight to the level of the speech disorder. The tool discussed in study F detects errors in a voice with the help of a hidden Markov model (HMM). The spoken phonemes by the client are compared with the target phoneme voice. The model can detect insertion, deletion and substitution. They also described a face tracker used to analyze the client’s nonverbal behavior by coordinating their eyes. Study H used a decision tree (DT) classifier for evaluating the correctness of speech samples. With this method, they managed to reach correct classification of the pronunciation of almost 93%. The authors then used this information to extend their phonological database. Other classifiers, such as KNN, random forest classifier and Adam’s neural network reached lower accuracy than 93%. Study M applied a three-layer ANN for speech recognition. Study W created a system that can suggest a helpful therapeutic plan for each client with fuzzy logic. The fuzzy expert system can recommend the needed follow-up actions, such as some exercises needed for a client based on several parameters. Study Y and Z used an ANN to generate a therapy plan, for which they used a multilayer perceptron. Study CC used CNN, which breaks the words into pieces and then analyses them. Study DD used a fuzzy logic algorithm to answer how frequent the therapy sessions should be, how long they should take and what types of exercises should be included. Study FF used three types of classifiers to enable speech recognition, namely, a multi-layer perceptron, an SVM and a logistic regression model with the help of MATLAB Toolboxes. Study HH used neural networks to detect the severity of the disorder.

**Figure 7:**
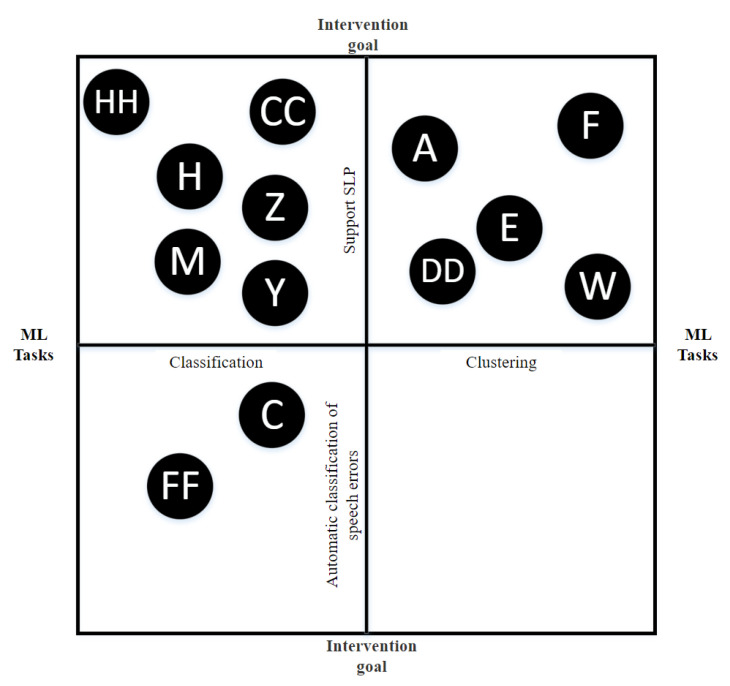
Bubblechart of ML approaches and OST goals.

Figure 7 shows the relationships between ML approaches and the OST goals. The figure shows that ML was only used for two of the five intervention goals, namely, “support the SLP” and “automatic classification of speech errors”. Not surprisingly, the two studies C and FF that had automatic classification as a goal only had classification as a ML task. For the intervention goal of supporting the SLP, both classification and clustering were used.

### 4.6. What Are the Properties of the Software Used for These Systems?

**Figure 8:**
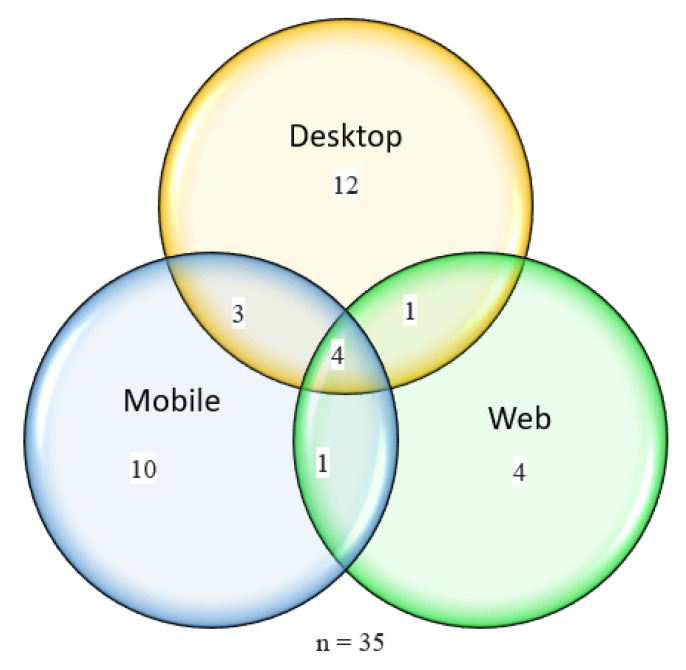
OST delivery types of primary studies.

We describe the delivery type and indicate whether it is designed for desktops, mobile phones, browsers or multiple formats. The supported platform for each piece of software can affect the number of users and its accessibility. If the software supports different platforms, the range of users might be many and more exhaustive. Our review shows that most of the systems are desktop-based, followed by mobile-based. Figure 8 shows the distribution of delivery types in numbers. Desktop only is the most popular one, with 34%, followed by mobile-only, with 29%.

### 4.7. Which Evaluation Approaches Have Been Used to Assess the Efficacy of the OST Systems?

Table 9 shows the evaluation approach that each of the studies used to evaluate its OST system. Overall, five main evaluation approaches were observed across all studies; the case study approach was more prevalent than the others. None of the studies adopted two or more approaches. Each evaluation approach is discussed below.

#### 4.7.1. Case Study

Sixteen studies tested the discussed OST system with a case study. All sixteen stated the sample size (see Appendix C), apart from study U. Study U mentioned that pilot tests with children with disabilities and typically developing children were done, but not the sample size. Study C first did a screening activity for six months with 356 5- to 9-year-old children. During this screening period, SLPs were asked to fill in individual reports for every child about the screening results. Children were assessed individually in a quiet room in their school setting by an SLP or an SLP graduate student. Each child had two different screening moments. Study G’s system evaluation was done throughout therapy sessions in seven weeks, with five 2- to 6-year-old children. Throughout the sessions, the SLP made notes about the improved receptive vocabulary of the children. The authors of study K tested their system with a within-subject study where children played two versions of the game. During the game, the children were asked to fill in surveys. The authors analyzed meta-data to identify differences in versions in the amount of speech practice completed. Study L consulted a technical evaluation by asking 30 IT professionals to fill in an evaluation form and rate the developed mobile application. In study M, the software was used for five months in the therapy at a school for the hearing impaired to improve the number of correctly pronounced vowels before and after five months of therapy. The tool from study O was evaluated in the therapy of speech disorders by performing a sensitivity test. The SLP noted that when the target sound was reached or not during the therapy, and there was a statistically significant improvement after therapy. A preliminary user study was done in study P to evaluate whether people would understand the concept of the OST application. As can be seen in Appendix C, only one of the five participants could be considered as a minor. However, the authors indicated that their OST system can also be used by young people; thus, we did not exclude it from our analysis. Study R tested their game with a 4-year-old and a 6-year-old and checked their test results. The drawback of this study was that apart from age, no additional information about the two children was given. After using the application, the participants were asked to fill in a questionnaire anonymously. The authors of study S conducted a pilot experiment consisting of two stages: a first one consisting of laboratory tests to determine the robot’s performance and a second stage to analyze the client’s response to the robot’s appearance. Study U evaluated the system in four families with one or more children with disabilities who were currently receiving speech therapy. The system in study V was validated through a pilot study with children diagnosed with apraxia of speech, together with their parents and SLPs. After a session of ten minutes, the children were asked several questions, and the SLP and parents were asked to fill in questionnaires. A similar procedure was conducted for the system in study X, where a pilot experiment was conducted. However, in this study, only the SLPs were asked to fill in the survey. Similarly, for study Y and study Z, a pilot experiment was conducted, during which SLPs were asked to execute evaluations with the help of an online tool. Study AA used three different methods of assessment. First, they asked SLPs to provide feedback on the application. Secondly, they asked three usability experts, such as a professor in computer engineering, to perform a heuristic evaluation. Finally, they performed user tests with a group of children with speech disorders and a group without. In study GG, twenty participants used the developed application and filled in a questionnaire to rate the usability afterward.

#### 4.7.2. Experimental

Five studies validated the discussed OST system with an experiment. The participants’ characteristics can be found in Appendix C. Study E evaluated the performances of the system with the help of forty 5- to 6-year-old children. Twenty children attended classical therapy sessions, and the other 20 used the developed system. Statistical tests were done to compare the difference between groups after 24 meetings. For investigating the performance of the system in study I, participants were asked to carry out tasks under the supervision of an SLP in a quiet room. During the experiment, responses were recorded by observation and video. For quantitative analysis, the total number of correct responses was calculated with the help of a language assessment tool. Additionally, the researchers asked the speech therapist whether they agreed with the performance score that was assigned by each tool to each participant. In study Q, one-to-one, 45 min sessions were performed with the participants and the SLP. Additionally, semi-structured interviews were conducted with both therapist and parents. Each session was examined by an observer who noted the interaction between the child and the SLP with conversational analysis. Study T administered the gaze targets of an experimental group and the control group. Based on the time interval for which the child was looking at the robot, the level of engagement was estimated. To test the effectiveness of the tool of study BB, an experiment was conducted in two schools for special education, considering objective measurements from the statistical analysis of the results stored by the tool and the subjective measurements from a therapy evaluation form for each user proposed by the therapist.

#### 4.7.3. Observational

Study B tested the system with two 5-year-old children. The researchers observed both sessions of the children with the SLP and gave a description of what they saw in the paper. The participants of study J were observed while executing a task with the systems. Usability tests were executed to evaluate the interaction of the participants while working directly with the system. Both studies mentioned the characteristics of their participants (Appendix C).

#### 4.7.4. Simulation-Based

The usability of the proposed application of study A was validated with a software test. Study D tested its modules on target datasets. Study H adopted and implemented the most extensive evaluation amongst all selected studies. The researchers evaluated the proposed system with a database that consisted of speech samples collected from 1114 evaluations with 1077 children, resulting in a database containing 93,576 audio samples. The game application of study CC was tested by using a Torgo Dataset that contained audio data of people with and without dysarthria. Study FF evaluated the performances of their trained algorithms using three experiments. For study EE, a proof-of-concept prototype was set up, which seemed to work fine but needs further clinical evaluation.

#### 4.7.5. Not Evaluated

Study F mentioned in the discussion that they are planning in the future to evaluate the proposed system. Likewise, study N explained at the end that their proposed application would be tested later. In study W, they mentioned that their system has been validated experimentally, but the authors did not provide further explanations on the experiment. Noteworthy for study HH is that the authors mentioned the procedures that are part of a testing stage but did not explain any further.

### 4.8. Which Evaluation Metrics Have Been Used to Gauge the Efficacy of the OST Systems?

**Figure 9:**
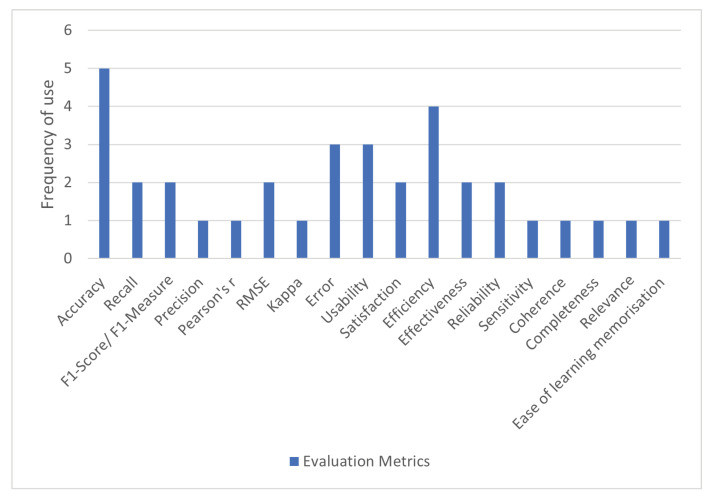
The evaluation metrics adopted by the selected articles.

The evaluation metrics that were encountered in the literature are displayed in Figure 9. Five articles did not evaluate the performances of their models, whereas some articles used multiple metrics. Table 10 provides an overview of the metrics found in the articles. The metrics that were used the most were accuracy and efficiency, which are explained in the next paragraph. The metrics used by the studies differed, as studies tested different aspects of their systems. Two main categories evaluation metrics were used, namely, machine learning-based (ML) and general evaluation metrics. Below, we discuss the metrics under the two main categories.

ML Evaluation Metrics:

- Accuracy describes the percentage of correctly predicted values.
- Recall is the proportion of all true positives predicted by the model divided by the total number of predicted values [15]. Recall=$\frac{TP}{TP+FN}$. TP = true positives. FN = false negatives.
- F1-score is a summary of both recall and precision (Russell and Norvig, 2010). F1−Score=2∗$\frac{Precision*Recall}{Precision+Recall}$.
- Precision calculates the proportion of correctly identified positives (Russell and Norvig, 2010). Precision=$\frac{TP}{TP+FP}$. TP = true positives. FP = false positives.
- Pearson’s r is a statistical method that calculates the correlation between two variables.
- Root-mean-square deviation (RMSE) calculates the difference between the predicted values and the observed values.
- $RMSE=\sqrt{\frac{\sum_{i=1}^{N}{\left(Predicted_{i}-Actual_{i}\right)}^{2}}{N}}$.
- Kappa is a method that compares the observed and expected values.
- Error refers to the average error of the system regarding its measures.

General Evaluation Metrics:

- Usability refers to the effectiveness, efficiency and satisfaction together [27].
- Satisfaction refers to how pleasant or comfortable the use of the application is [43,47].
- Efficiency refers to the resources spent to achieve effectiveness, such as time to complete the task, the mistakes made and difficulties encountered [27].
- Effectiveness refers to the number of users that can complete the tasks without quitting [27].
- Reliability refers to the level at which the application responds correctly and consistently regarding its purpose [29].
- Sensitivity is the level at which the tool can discriminate the proper pronunciations from the wrong ones [32].
- Coherence specifies whether the exercises selected by the system are appropriate for the child, according to their abilities and disabilities [41].
- Completeness determines if the plans recommended by the expert system are complete and takes into account the areas in which the child should be trained to develop specific skills (according to the child’s profile) [41].
- Relevance determines if each exercise’s specificity is appropriate for the child [41].
- Ease of learning memorization looks at how easy it is for the user to perform simple tasks using the interface for the first time [47].

## 5. Discussion and Limitations of the Review

The review included studies that looked at various types of disorders, outcome measures and levels of evidence. There seems to be a need for authors that are both experts in the communication disorders field and in the software engineering field. However, no publication has been found that addresses both of these concerns clearly. Even though papers and definitions were checked by a certified SLP, the different categorization systems and broad variety of disorders make it difficult to put all the systems in a clear group that everybody would agree with. Thus, one of the challenging aspects of this review was the high heterogeneity among the discussed papers, as in the study from Chen et al. [11], making it challenging to draw general conclusions. The studies showed broad variations in study designs and methodological quality. Some experimental studies had small sample sizes—for example, only five participants—making it risky to draw any overall conclusions on the systems, as the OST Systems were proposed for various communication disorders. The classification of communication disorders is somewhat complex and versatile, and although we provided a brief summary in the background (Section 2), it is essential to realize that each communication disorder has its own therapy approach. Some of the developed systems were targeted at SSDs, which is a rather broad category. We also found that many papers lacked sufficient details about the architecture used, which can be useful for researchers as inspiration for building their own systems. The findings from this study could have suffered from some limitations, which should be acknowledged when interpreting the results. A common threat to the validity of an SLR can be the so-called publication bias. We tried to minimize this limitation by developing a research protocol and writing down our methodology on a detailed level. Even though the search query used to perform the SLR was rather extensive, there is always a risk that some relevant literature is overlooked. In an ideal case, all the 4481 articles found within the query would be scanned more thoroughly, and not just their titles and abstracts. However, this would have been not feasible regarding the given time for this research. The data extraction was conducted as objectively as possible, yet there is a chance that some details were overlooked. To validate the extraction process, the second and third author did random cross-checks. Future research could look at how many of the researched OST systems are being used. Some of the studies reported that the discussed systems were also introduced to the market. However, it would be interesting to see if some of the systems are still being used. We also acknowledge that other evaluation metrics which are useful were not reported in our study. Metrics that could discern if the child’s speech has improved are not used by researchers who developed tools and these kind of metrics were missing from our study. It would be interesting to examine or propose more practical metrics that assesses the performances of children who use these developed tools.

## 6. Conclusions

Speech therapy is a very essential procedure for children with communication disorders. However, not all children with communication disorders have access to the limited number of speech–language pathologists (SLP). Fortunately, several online speech therapy (OST) systems have been designed and proposed. Previous systematic reviews on OST systems for children with speech sound disorders (SSDs) are limited and discuss a wide variety of features. Through a systematic literature review, this paper examined currently existing automated speech therapy programs that have been discussed in prior literature. Eight research questions were set up to obtain further information on the existing OST systems and to obtain a deeper understanding of the current challenges of the OST systems. Out of the 4481 papers found by our search strategy, 35 of the papers primarily focused on OST systems for children with speech disorders. Our analysis shows that there is a wide variety of systems that have already been developed. The main goal of most designed OST systems was to support the SLP in their tasks. Systems are available in different languages and for different target disorders. It is challenging to understand how some of these OST systems are set up, as most studies did not describe a reference architecture (RA). The studies that mainly did used a client–server approach, which provides the clients with speech therapy services with the help of a database. Additionally, the number of studies that adopted and used machine learning techniques was lower than the number that did not. This finding explains why there are so many OST systems designs, yet only a few are eventually developed and implemented for practical use.

## Figures and Tables

**Table 1:** Process of paper selection.

Source	Before	After Abstract Screening	After Applying the Selection Criteria	After Quality Assessment
IEEE	930	130	10	9
Scopus	2704	160	18	16
Web of Science	619	90	6	6
ACM	228	19	5	4
**Total**	4481	399	39	35

**Table 2:** Study selection criteria.

No.	Criteria
1	The full text is unavailable.
2	The duplicate publication is already found in a different repository.
3.	Article language is not in English.
4.	The article is not relevant or related to child speech therapy.
5.	The article is not a primary study.
6.	The article is not peer-reviewed.
7.	The article only focuses on speech recognition techniques and not on therapy.

**Table 3:** Evaluation standards. The answer to each question scores the quality of the paper.

Nr.	Criteria	Yes (2)	Partial (1)	No (0)
1	Are the aims of the study clearly stated?			
2	Are the scope and context of the study clearly defined?			
3	Is the proposed solution clearly explained and validated by an empirical study?			
4	Are the variables used in the study likely to be valid and reliable?			
5	Is the research process documented adequately?			
6	Are all study questions answered?			
7	Are the negative findings presented?			

**Table 4:** Final articles selected.

Nr.	Reference	Title
A	[20]	Tingog: Reading and Speech Application for Children with Repaired Cleft Palate
B	[21]	A serious game for speech disorder children therapy
C	[22]	The BioVisualSpeech Corpus of Words with Sibilants for Speech Therapy Games Development
D	[23]	Advanced Information Technology—Support of Improved Personalized Therapy of Speech Disorders
E	[2]	TERAPERS -Intelligent Solution for Personalized Therapy of Speech Disorders
F	[24]	An automated speech-language therapy tool with interactive virtual agent and peer-to-peer feedback
G	[25]	Android based Receptive Language Tracking Tool for Toddlers.
H	[1]	Towards a speech therapy support system based on phonological processes early detection.
I	[26]	Assessing comprehension of spoken language in nonspeaking children with cerebral palsy: Application of a newly developed computer-based instrument
J	[27]	AppVox: An Application to Assist People with Speech Impairments in Their Speech Therapy Sessions
K	[28]	Apraxia world: A Speech Therapy Game for Children with Speech Sound Disorders
L	[29]	Speak App: A Development of Mobile Application Guide for Filipino People with Motor Speech Disorder
M	[30]	Speech technologies in a computer-aided speech therapy system
N	[31]	ChilDiBu—A Mobile Application for Bulgarian Children with Special Educational Needs
O	[32]	Audiovisual Tools for Phonetic and Articulatory Visualization in Computer-Aided Pronunciation Training
P	[33]	Building on Mobile towards Better Stuttering Awareness to Improve Speech Therapy
Q	[34]	Pictogram Tablet: A Speech Generating Device Focused on Language Learning
R	[35]	Measuring performance of children with speech and language disorders using a serious game
S	[36]	A robotic assistant to support the development of communication skills of children with disabilities
T	[37]	Evaluating a multi-avatar game for speech therapy applications
U	[38]	Secure telemonitoring system for delivering telerehabilitation therapy to enhance children’s communication function to home
V	[39]	Architecture of an automated therapy tool for childhood apraxia of speech
W	[40]	Translation of the Speech Therapy Programs in the Logomon Assisted Therapy System.
X	[41]	An educational platform based on expert systems, speech recognition, and ludic activities to support the lexical and semantic development in children from 2 to 3 years.
Y	[3]	SPELTA: An expert system to generate therapy plans for speech and language disorders.
Z	[42]	SPELTA-Miner: An expert system based on data mining and multilabel classification to design therapy plans for communication disorders.
AA	[43]	The AppVox mobile application, a tool for speech and language training sessions
BB	[10]	A prelingual tool for the education of altered voices
CC	[9]	A Game Application to assist Speech Language Pathologists in the Assessment of Children with Speech Disorders
DD	[44]	End-User Recommendations on LOGOMON - a Computer Based Speech Therapy System for Romanian Language
EE	[45]	Multimodal Speech Capture System for Speech Rehabilitation and Learning
FF	[46]	Tabby Talks: An automated tool for the assessment of childhood apraxia of speech
GG	[47]	AACVOX: mobile application for augmentative alternative communication to help people with speech disorder and motor impairment
HH	[5]	An Online Expert System for Diagnostic Assessment Procedures on Young Children’s Oral Speech and Language
II	[48]	E-inclusion technologies for the speech handicapped

**Table 5:** OST Features.

Feature	Study	Total Number
Audio feedback	C, E, J, W, AA, BB	6
Emotion Screening	P	1
Error Detection	V, AA, CC, EE, FF, HH, II	7
Peer-to-peer feedback	F, K	2
Recommendation strategy	H, S, W, Y, Z	5
Reporting	D, S, V, W, X, Y, Z, AA, BB, CC, DD, FF, GG, HH	14
Speech Recognition	A, H, M, O, S, V, X, BB, CC, EE, II	11
Text-to-speech	A, S, GG, II	4
Textual feedback	F, J, II, CC, FF	5
User Data Management	S, X, Y, Z, DD, II	6
User Record voice	E, Q, U, V, W, CC, EE, FF, GG, II	10
Virtual 3D model	E, O, W, DD, EE	5
Visual feedback	C, EE, II	3
Voice commands	R, S	2

**Table 6:** Classification of communication disorders.

Classification	Study
Communication disorder	S, X, Z,
Speech disorder	A, C, D, E, H, K, L, N, P, Q, V, W, BB, CC, DD, EE, FF, GG, II
Language disorder	B, F, I, J, R, T, U, Y, AA, HH
Hearing disorder	G, M, O

**Table 7:** Overview of architectures of OST systems.

Adopted Architecture Approach	Study
client–server system	D, F, H, L, P, U, V, DD, HH, II
Repository pattern	T, CC
Layered approach	S, X, Y, Z
Standalone system	A
Pipe-and-Filter Architecture	E, W, FF

**Table 8:** Overview of adopted ML approaches.

Nr.	ML Types	ML Tasks	Algorithms	Application	Adopted Dataset
A	Unsupervised	Clustering	Not mentioned	Speech Recognition	Not mentioned
C	Supervised	Classification	Convolutional Neural Networks (CNN) Hidden-Markov Model	Speech Recognition	The database contains reading aloud recordings of 284 children. The corpus contains reading aloud recordings from 510 children.
E	Unsupervised	Clustering	Not mentioned	Generate a therapy plan	Not mentioned
F	Unsupervised	Clustering	Hidden Markov Model	Time prediction	Not mentioned
H	Supervised	Classification	Decision Tree Neural Network Support Vector Machine k-Nearest Neighbor Random Forest	Speech classification	A Phonological Knowledge Base containing speech samples collected from 1114 evaluations performed with 84 Portuguese words.
M	Supervised	Classification	Artificial Neural Networks (ANN)	Speech recognition	The authors refer to a large speech database, but no further details are given.
W	Unsupervised	Clustering	Not mentioned	Generate a therapy plan	Not mentioned
Y	Supervised	Classification	Decision Tree Artificial Neural networks	Generate a therapy plan	Not mentioned
Z	Supervised	Classification	Artificial Neural Networks	Generate a therapy plan	Database of thousands of therapy strategies.
CC	Supervised	Classification	Convolutional Neural Networks (CNN)	Speech to Text	TORGO Dataset that contains audio data of people with dysarthria and people without dysarthria.
DD	Unsupervised	Clustering	Not Mentioned	Emotion recognition	Not applicable
FF	Supervised	Classification	Artificial Neural Network (ANN) Logistic regression Support Vector Machine	Speech recognition	A dataset with correctly-pronounced utterances from 670 speakers.
HH	Supervised	Classification	Neural Networks	Detect disorder	Not mentioned

**Table 9:** Approaches to evaluation of the literature.

Evaluation Approach	Study
Case Study	C, G, K, L, M, O, P, R, S, U, V, X, Y, Z, AA, GG
Experimental	E, I, Q, T, BB, DD
Not evaluated	F, N, W, HH
Observational	B, J
Simulation-based	A, D, H, CC, EE, FF, II

**Table 10:** Evaluation metrics used in the selected literature.

Metrics	Study
**ML Evaluation Metrics**	
Accuracy	H, M, Z, CC, FF
Recall	H, FF
F1-Score/ F1-Measure	H, FF
Precision	FF
Pearson’s r	I
RMSE	H, EE
Kappa	I
Error	H, FF, II
**General Evaluation Metrics**	
Usability	A, L, GG
Satisfaction	AA, GG
Efficiency	L, AA, DD, GG
Effectiveness	J, AA
Reliability	L, T
Sensitivity	O
Coherence	X
Completeness	X
Relevance	X
Ease of learning memorization	GG

## Data Availability

The data extraction was done with the help of a Microsoft Excel spreadsheet which is freely available online Available online: https://bit.ly/3Fv7qDp (accessed on 1 November 2022).

## Appendix A. Quality Assessment Form

**Table A1:** Quality assessment score.

Reference	Q1. Are the Aims of the Study Clearly Stated?	Q2. Are the Scope and Context of the Study Clearly Defined?	Q3. Is the Proposed Solution Clearly Explained and Validated by an Empirical Study?	Q4. Are the Variables Used in the Study Likely to Be Valid and Reliable?	Q5. Is the Research Process Documented Adequately?	Q6. Are All Study Questions Answered?	Q7. Are the Negative Findings Presented?	TOTAL SCORE
[20]	1	2	2	2	2	2	1	12
[21]	1	2	1	1	1	1	1	8
[22]	2	2	2	2	2	2	1	13
[23]	2	2	0	2	1	2	2	11
[2]	2	2	2	2	2	2	2	14
[24]	1	2	0	1	1	1	2	8
[25]	1	1	2	1	1	2	0	8
[1]	1	2	2	2	2	2	1	12
[26]	1	2	2	2	2	2	2	13
[27]	2	2	2	2	2	1	2	13
[49]	1	2	0	1	1	1	1	7
[28]	2	2	2	2	2	2	2	14
[29]	1	2	2	2	1	1	1	10
[30]	1	2	2	1	1	2	1	10
[31]	1	2	0	2	2	1	2	10
[32]	2	2	2	2	2	2	2	14
[50]	1	1	0	1	0	1	1	5
[33]	2	2	1	1	1	2	2	11
[34]	1	1	2	2	2	1	2	11
[35]	2	2	1	1	1	2	2	11
[36]	2	2	2	1	1	1	1	10
[37]	2	1	2	2	2	1	2	12
[38]	1	2	0	1	2	2	2	10
[39]	2	2	2	2	2	2	2	14
[40]	2	2	1	2	2	1	0	10
[41]	1	2	2	2	2	1	0	10
[3]	2	2	2	2	2	2	2	14
[42]	2	2	2	2	2	2	2	14
[43]	2	2	2	2	2	2	2	14
[10]	2	2	2	2	2	2	2	14
[9]	2	1	1	1	2	2	2	11
[44,45]	2	1	0	1	2	2	2	10
[46]	2	2	2	2	2	2	2	14
[47]	2	2	2	1	1	1	2	11
[5]	2	2	0	1	2	2	0	9
[48]	1	1	1	1	2	2	2	10
[51]	2	1	0	1	0	2	0	6
[52]	1	1	0	1	1	1	1	6

## Appendix B. Data Extraction Form

**Table A2:** Data extraction form.

#	Extraction Element	Contents
**General Information**		
1	ID	
2	Reference	
3	SLR Category	Include vs Exclude
4	Title	The full title of the article
5	Year	The publication year
6	Repository	ACM, IEEE, Scopus, Web of Science
7	Type	Journal vs article
**Demographic and methodological characteristics of the included studies**		
8	Intervention target	
9	Disorder (Target group)	
10	Target language	
11	Sample Size	
12	Participant characteristics	
13	Evaluation	
14	Outcome measure	
**Technological elements of OST systems**		
15	OST Name	
16	System objective	
17	Architecture design	
18	ML approach	
19	OST Technology details	

## Appendix C. Sample Size Characteristics of Experimental Studies

**Table A3:** Sample size characteristics of experimental studies.

Author	Sample Size	Participant Characteristics
B	2	(1M, 1F): One 5-year-old Turkish speaking boy with no language/speech problem and one 5-year-old Turkish speaking girl with a language disorder because of hearing impairment.
C	356	5 to 9 years old Portuguese speaking children
E	40	5 to 6 years old Romanian speaking children, boys and girls with difficulties in pronunciation of R and S sounds.
G	5	2 to 6 years old English speaking children with hearing impairment.
H	1077	3 to 8 years old Portuguese speaking children.
I	60	(22M, 20F): 42 14 to 60 years old Dutch-speaking children and adults without disabilities. (9M, 9F): 18 19 to 75 months old. Dutch-speaking children with severe cerebral palsy.
J	4	(2M, 2F): 8 to 10 years old Portuguese speaking children
K	21	(13M, 1F): fourteen 4 to 12 year old with diagnosed SSDs ranging from mild to severe (7 motor-speech and 7 phonological impairments). (4M, 3F): seven 5 to 12 years old children typically developing
L	30	IT professionals
M	10	Deaf, hard of hearing, implanted children and those who had a speech impediment.
O	11	(6M, 5F): 5,2 to 6,9 years old German-speaking children suffering from a specific articulation disorder, i.e., [s]-misarticulation
P	5	Portuguese speaking. 2 females (30 and 46 years) and 3 males (ages: 13, 33, 36). The younger participant is the only one doing speech therapy.
Q	18	16 boys and 2 girls were recruited from three psychology offices. Their mean age was 10.54 years (range 2–16; std 4.34).
R	1	A 4-year-old and a 6-year-old.
S	32	Children of regular schools
T	12	Italian speaking children
U	Unknown	Children with disabilities and typically developing children.
V	8	(3M, 1F): 3 to 7 years old children clinically diagnosed with apraxia of speech.
X	22	2-year-old children
Y	53	Children with different types of disabilities and cognitive ages from 0 to 7 years
Z	53	Children with different types of disabilities and cognitive ages from 0 to 7 years
AA	35	(13M, 7F): 20 7 to 10 years old children with no speech or language impairments (9M, 6F): 15 7 to 9 year old with speech and language impairments
BB	27	11 to 34 years old children and adults with mild to severe mental delay or a communication disorder.
DD	143	43 parents and 100 teachers (kindergarten and primary school
FF	Unknown	Children and adults with different levels of dysarthria.
GG	20	(11M, 9F): 15 to 55 years old CP volunteers with speech difficulties and motor impairment.
II	14	(7M, 7F): 11 to 21 years old children and adults with physical and psychical handicaps like cerebral palsy, Down’s syndrome and similar impairments.
